# Supplementary material for: Semantic search using protein large language models detects class II microcins in bacterial genomes
Source: bioRxiv. 2023 Nov 15:2023.11.15.567263. Preprint. [Version 1] doi: 10.1101/2023.11.15.567263 (PMC10680697; doi:10.1101/2023.11.15.567263)
Supplement: Supplement 1 [file media-1.pdf]

Supplementary Figures and Files

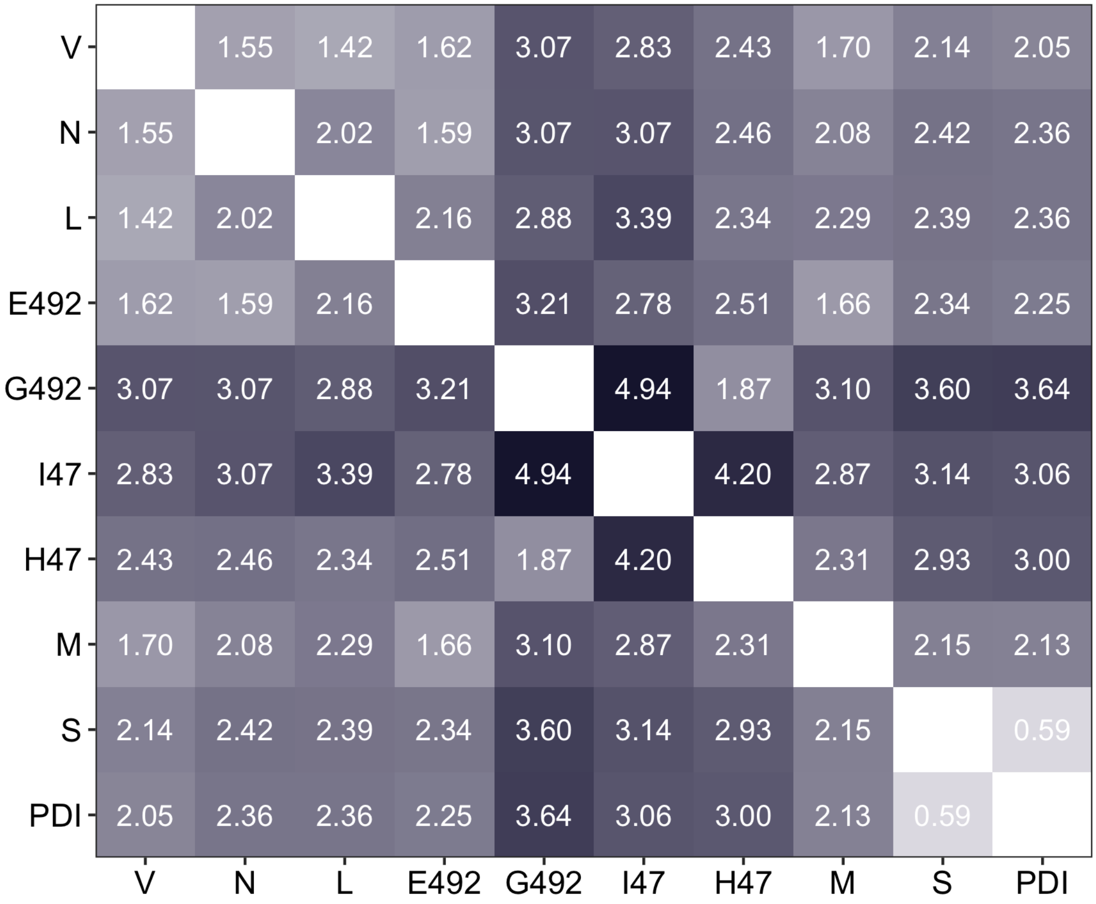

Supplementary Figure S1: Embedding distance between all pairs of the ten known microcins.

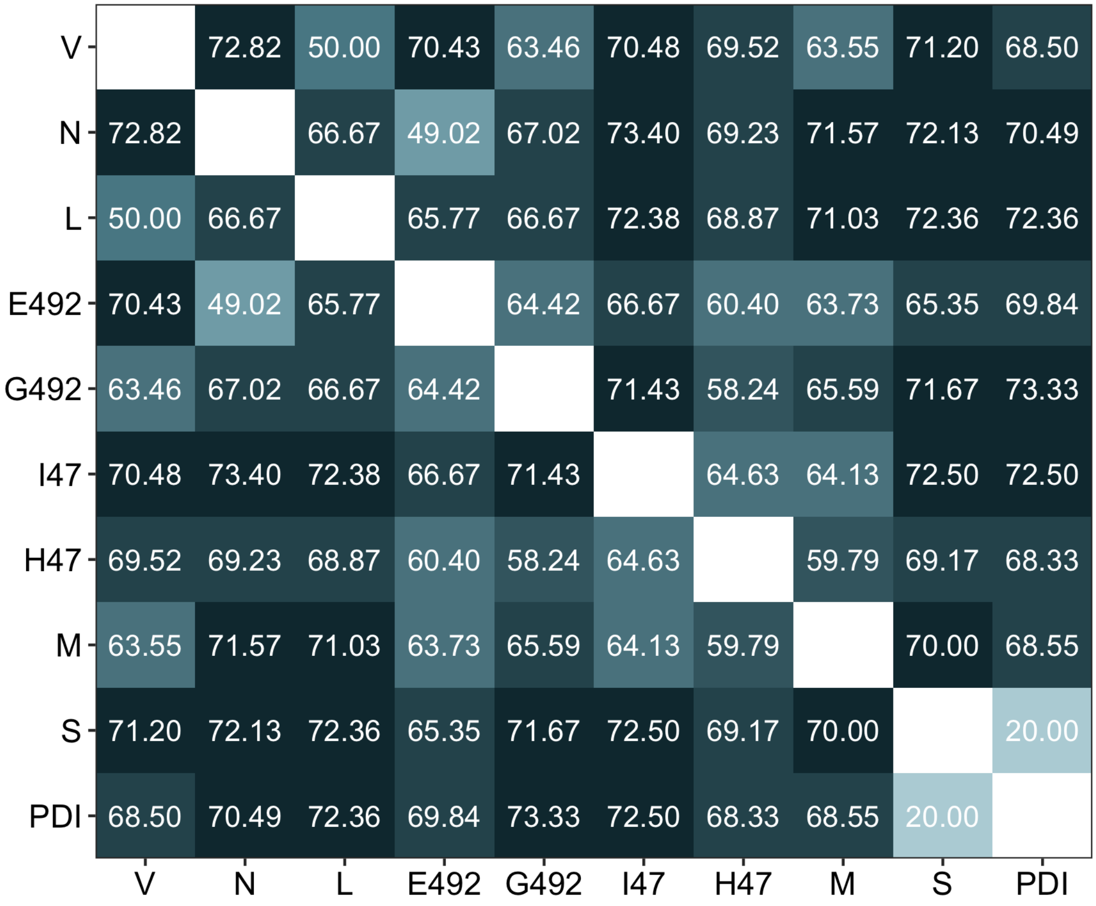

Supplementary Figure S2: Percent sequence divergence between all pairs of the ten known microcins.

Supplementary File S1: Information about the dataset of 25 *E. coli* genomes. Contains the columns name, strain_name, accenssion_number, strain_category, phylogroup, Genome_ID, microcin_hit_count, CvaB_hit_count.

Supplementary File S2: Information about the dataset of *Enterobacter* genomes. Contains the columns accession, ncbi_organism_name, cinful_CvaB_found (indicates whether a CvaB was previously found by cinful—the value is TRUE for all genomes in this set), cinful_microcins_found.

Supplementary File S3: Information about the dataset of *Klebsiella* genomes. Contains the columns accession, ncbi_organism_name, cinful_microcins_found (number of microcins found by cinful), cinful_CvaB_found (indicates whether a CvaB was previously found by cinful—the value is TRUE for all genomes in this set).

Supplementary File S4: Cinful microcin and CvaB hits from the Touchon dataset. Contains the columns name, strain_name, accession_number, strain_category (origin of the bacteria), phylogroup, Genome_ID, microcin_hit_count (how many microcins were detected by Cinful), CvaB_hit_count (the number of microcin exporter proteins Cinful detected), Assembly (accession number for retrieval from NCBI).

Supplementary File S5: Cinful results for 40 water-sourced and extra-intestinal genomes from the Touchon dataset. Contains the columns name, strain_name, accession_number, strain_category (origin of the bacteria), phylogroup, Genome_ID, microcin_hit_count (how many microcins were detected by Cinful), CvaB_hit_count (if Cinful detected a microcin exporter protein), Assembly (accession number for retrieval from NCBI).

Supplementary File S6: Amino acid sequences of the ten known microcins, provided in FASTA format.

Supplementary File S7: Alignment of embedding hits collected from 25 *E. coli* genomes.

Supplementary File S8: Putative microcins found in the *E. coli*, *Enterobacter*, and *Klebsiella* datasets.

Supplementary File S9: Alignment of embedding hits collected from 44 *Enterobacter* genomes.

Supplementary File S10: Alignment of embedding hits collected from 46 *Klebsiella* genomes.
